# Rabeprazole Promotes Vascular Repair and Resolution of Sepsis-Induced Inflammatory Lung Injury through HIF-1α

**DOI:** 10.3390/cells11091425

**Published:** 2022-04-22

**Authors:** Colin E. Evans, Yi Peng, Maggie M. Zhu, Zhiyu Dai, Xianming Zhang, You-Yang Zhao

**Affiliations:** 1 Program for Lung and Vascular Biology, Stanley Manne Children’s Research Institute, Ann & Robert H. Lurie Children’s Hospital of Chicago, Chicago, IL 60611, USA; yi.peng@northwestern.edu (Y.P.); mengqizhu2021@u.northwestern.edu (M.M.Z.); zhiyudai@arizona.edu (Z.D.); xiazhang@luriechildrens.org (X.Z.); 2 Section for Injury Repair and Regeneration Research, Stanley Manne Children’s Research Institute, Ann & Robert H. Lurie Children’s Hospital of Chicago, Chicago, IL 60611, USA; 3 Department of Pediatrics, Division of Critical Care, Northwestern University Feinberg School of Medicine, Chicago, IL 60611, USA; 4 Department of Medicine, Division of Pulmonary and Critical Care Medicine, Northwestern University Feinberg School of Medicine, Chicago, IL 60611, USA; 5 Feinberg Cardiovascular and Renal Research Institute, Northwestern University Feinberg School of Medicine, Chicago, IL 60611, USA; 6 Department of Pharmacology, Northwestern University Feinberg School of Medicine, Chicago, IL 60611, USA

**Keywords:** ARDS, FoxM1, HIF-1α, hypoxia-inducible factor, inflammation, rabeprazole, sepsis

## Abstract

There are currently no effective treatments for sepsis and acute respiratory distress syndrome (ARDS). The repositioning of existing drugs is one possible effective strategy for the treatment of sepsis and ARDS. We previously showed that vascular repair and the resolution of sepsis-induced inflammatory lung injury is dependent upon endothelial HIF-1α/FoxM1 signaling. The aim of this study was to identify a candidate inducer of HIF-1α/FoxM1 signaling for the treatment of sepsis and ARDS. Employing high throughput screening of a library of 1200 FDA-approved drugs by using hypoxia response element (HRE)-driven luciferase reporter assays, we identified Rabeprazole (also known as Aciphex) as a top HIF-α activator. In cultured human lung microvascular endothelial cells, Rabeprazole induced HIF1A mRNA expression in a dose-dependent manner. A dose-response study of Rabeprazole in a mouse model of endotoxemia-induced inflammatory lung injury identified a dose that was well tolerated and enhanced vascular repair and the resolution of inflammatory lung injury. Rabeprazole treatment resulted in reductions in lung vascular leakage, edema, and neutrophil sequestration and proinflammatory cytokine expression during the repair phrase. We next used *Hif1a*/*Tie2*Cre knockout mice and *Foxm1*/*Tie2*Cre knockout mice to show that Rabeprazole promoted vascular repair through HIF-1α/FoxM1 signaling. In conclusion, Rabeprazole is a potent inducer of HIF-1α that promotes vascular repair and the resolution of sepsis-induced inflammatory lung injury via endothelial HIF-1α/FoxM1 signaling. This drug therefore represents a promising candidate for repurposing to effectively treat severe sepsis and ARDS.

## 1. Introduction

There are currently no effective treatments for severe sepsis and acute respiratory distress syndrome (ARDS) [1]. Sepsis-induced inflammatory lung injury is characterized by pulmonary edema, microvascular leakage, and enhanced inflammatory cytokine expression [1,2]. Endothelial barrier repair is necessary to restore vascular homeostasis and tissue fluid balance after inflammatory lung injury [1,2,3]. Meanwhile, the extent of microvascular leakage can determine the outcome of sepsis in animals and humans [3,4,5,6,7,8,9,10]. Targeting of endothelial repair mechanisms that restore endothelial barrier integrity therefore represents a promising therapeutic option for the treatment of sepsis-induced inflammatory lung injury [11,12,13,14,15]. In other words, stimulation of the molecular pathways responsible for endothelial recovery and vascular repair could improve clinical outcomes in patients with sepsis/ARDS [11,12,13,14,15].

The adaptive responses to hypoxia and ischemia are controlled by hypoxia-inducible factors (HIFs) [16,17,18,19]. These heterodimeric proteins are made up of an oxygen-labile α subunit and a constitutively expressed β subunit [20,21,22]. Under normoxia, the α subunit is hydroxylated by the HIF prolyl hydroxylases (PHD1-3) and is then recognized by the von Hippel-Lindau (VHL) protein, resulting in proteasomal degradation by the ubiquitin-protein ligase complex [23,24]. Under hypoxia, however, HIF-α degradation is inhibited, leading to the accumulation of HIF-α in the nucleus, and subsequent formation of the HIF complexes with the HIF-β subunit [20,21,22]. The HIF complex then binds to the hypoxia response elements (HREs) of its target genes and controls the expression of genes critical for the adaptive responses to hypoxia and ischemia [16,17,18,19]. There are three isoforms of the α-subunit (i.e., HIF-1α, 2α, 3α) and one β subunit [25,26,27]. Our recent study showed that HIF-1α in endothelial cells (ECs) is responsible for vascular repair and the resolution of inflammatory lung injury [6]. Following sepsis challenge, HIF-1α expression was rapidly increased in lung vascular ECs to mediate vascular repair and the resolution of inflammatory lung injury, while mice with EC-specific disruption of *Hif1a* demonstrated impaired vascular repair, persistent inflammation, and increased mortality compared with wild type (WT) littermates [6]. Furthermore, transient expression of the reparative transcriptional factor, forkhead box M1 (FoxM1), in ECs resulted in the restoration of vascular repair and improved survival [4,6,28]. These studies together revealed the essential role of endothelial HIF-1α in driving post-sepsis vascular repair via FoxM1. Therapeutic activation of HIF-1α signaling and HIF-1α/FoxM1-dependent vascular repair therefore represents a putative therapeutic strategy for the treatment of inflammatory vascular diseases, such as sepsis and ARDS.

The first aim of this study was to identify potential HIF-1α inducers from a library of FDA-approved drugs. The second aim was to assess the impact of one of the identified drugs, Rabeprazole, on vascular repair and the resolution of inflammation in a mouse model of sepsis-induced inflammatory lung injury. The final aim was to determine whether Rabeprazole improves vascular repair and the resolution of sepsis-induced inflammatory lung injury via HIF-1α and FoxM1. Our studies together identify Rabeprazole as a candidate therapy for the treatment of ALI/ARDS.

## 2. Materials and Methods

### 2.1. Mice

Male and female C57BL/6 WT mice (aged 10–16 weeks) were used throughout the studies. For studies of conditional *Hif1a* or *Foxm1* knockout (KO) in ECs, *Hif1a* or *Foxm1* KO was driven by the *Tie2*Cre recombinase as previously described [6]. Briefly, *Tie2*Cre-expressing male mice were bred with female *Hif1a* floxed or *Foxm1* floxed mice to generate experimental *Hif1a/Tie2*Cre^−^ (WT) and *Hif1a/Tie2*Cre^+^ (KO) or *Foxm1/Tie2*Cre^−^ (WT) and *Foxm1/Tie2*Cre^+^ (KO) pups. Littermate *Tie2*Cre^−^ mice served as WT controls. The experiments were conducted according to NIH guidelines on the use of laboratory animals. The animal care and study protocols were approved by the Institutional Animal Care and Use Committees of Northwestern University Feinberg School of Medicine.

### 2.2. Mouse Model of Endotoxemia Sepsis

To induce endotoxemia sepsis, mice received lipopolysaccharide (LPS, 3 mg/kg, i.p., Santa Cruz, Dallas, TX, USA) as previously described [4,5]. Rabeprazole was administered orally in phosphate-buffered saline (PBS) at the indicated times and dosages.

### 2.3. High Throughput Screening for HIF Inducers

Stable 786-O cells transfected with HRE or SV40 promoter luciferase plasmid were seeded in 384-well plates (500 cells/well). Drugs were dissolved in DMSO and added to the wells at 24 h after cell seeding. At 48 h later, steadylite plus buffer (PerkinElmer, Waltham, MA, USA) was added to the plate and mixed. Following incubation at room temperature for 15 min to allow for complete cell lysis and signal generation, luminescence signal was measured using a fluorescence plate reader (Tecan, Baldwin Park, CA, USA). The drug screening was carried out at the Northwestern High Throughput Analysis Laboratory.

### 2.4. Primary Culture of Human Lung Microvascular ECs (HLMVECs)

Human lung microvascular ECs (HLMVECs, Lonza, Houston, TX, USA) were seeded into 6-well plates (300,000 cells/well) with complete growth medium. At 48 h after cell seeding when the cells reached 80% confluency, the cells were changed to basal medium supplemented with 2% FBS for 10 h and then treated with the indicated doses of Rabeprazole. Cells were collected for molecular analyses at 18 h post-treatment.

### 2.5. Assessment of Lung Edema and Vascular Permeability

Lung edema was assessed by measurement of lung wet–dry ratio as previously described [6,29]. Lung vascular permeability was assessed by measurement of lung Evans blue-conjugated albumin (EBA) flux as previously described [6,29]. Briefly, EBA (20 mg/kg, r.o.) was injected 50 minutes before lungs were perfused with PBS, blotted dry, and weighed. Lungs were then homogenized in 0.5 mL PBS and incubated with 1 mL formamide for 18 h at 60 °C. Lung homogenates were centrifuged at 21,000× *g* for 10 min. Optical density of the supernatant was determined at 620 nm and 740 nm.

### 2.6. Measurement of Lung Myeloperoxidase (MPO) Activity

PBS-perfused lungs were homogenized in 0.5 mL of 50 mM phosphate buffer and then centrifuged at 21,000 g for 15 min at 4 °C. The pellets were resuspended in phosphate buffer with 0.5% hexadecyl trimethylammonium bromide and subjected to a freeze–thaw cycle. Subsequently, the homogenates were centrifuged at 21,000× *g* for 15 min at 4 °C. Following addition of 34 μL of sample to 10 μL of O-dianisidine dihydrochloride (16.7 mg/mL) and 50 μL of H_2_O_2_ (0.015% *v*/*v*) in 1 ml of phosphate buffer, absorbance was measured at 460 nm every 20 s for 3 min.

### 2.7. Molecular Analyses

For quantification of mRNA expression of proinflammatory cytokines, HIF-1α and FoxM1, RNA was isolated from cultured ECs using the RNeasy Mini kit (Qiagen, Germantown, MD, USA) with DNase I digestion or from lung tissues using Trizol reagent (Invitrogen, Waltham, MA, USA) followed by cleaning with the RNeasy Mini kit (Qiagen, Germantown, MD, USA) including DNase I digestion. Following reverse transcription, qPCR analysis was performed using the ABI ViiATM 7 real-time PCR system (Thermo Fisher Scientific, Waltham, MA, USA) with SYBR Green master mix. Nucleotide primer sequences were as follows: human *HIF1A*, 5′-TTACAGCAGCCAGACGATCATG-3′ (forward) and 5′-TGGTCAGCTGTGGTAATCCACT-3′ (reverse); with human *18S rRNA*, 5′-TTCCGACCATAAACGATGCCGA-3′ (forward) and 5′-GACTTTGGTTTCCCGGAAGCTG-3′ (reverse) as control; mouse *Cdh5*, 5′-GGCCTAAGTGTCTCCTTGATTC-3′ (forward) and 5′-TGGGTGAGAAGTATGGTGACTG-3′ (reverse); mouse *Foxm1*, 5′-CACTTGGATTGAGGACCACTT-3′ (forward) and 5′-GTCGTTTCTGCTGTGATTCC-3′ (reverse); mouse *Hif1a*, 5′-TGATGTGGGTGCTGGTGTC-3′ (forward) and 5′-TTGTGTTGGGGCAGTACTG-3′ (reverse); mouse *Il1b*, 5′-AACCTGCTGGTGTGTGACGTTC-3′ (forward) and 5′-CAGCACGAGGCTTTTTTGTTGT-3′ (reverse); mouse *Il6*, 5′-TCCAGTTGCCTTCTTGGGACTG-3′ (forward) and 5′-AGCCTCCGACTTGTGAAGTGGT-3′ (reverse); mouse *Nfkb1*, 5′-ATGGCAGACGATGATCCCTAC-3′ (forward) and 5′-TGTTGACAGTGGTATTTCTGGTG-3′ (reverse); mouse *Pecam1*, 5′-GAGCCCAATCACGTTTCAGTT-3′ (forward) and 5′-TCCTTCCTGCTTCTTGCTAGCT-3′ (reverse); mouse *Tlr4*, 5′-ATGGCATGGCTTACACCACC-3′ (forward) and 5′-GAGGCCAATTTTGTCTCCACA-3′ (reverse); mouse *Vegfa*, 5′-GCACATAGAGAGAATGAGCTTCC-3′ (forward) and 5′-CTCCGCTCTGAACAAGGCT-3′ (reverse); with mouse *Cypa*, 5′-CTTGTCCATGGCAAATGCTG-3′ (forward) and 5′-TGATCTTCTTGCTGGTCTTGC-3′ (reverse) as control. Mouse and human gene expression were normalized to mouse *Cypa* and human *18S rRNA*, respectively.

For quantification of HIF-1α protein levels, lungs were analyzed by Western blotting as previously described [6]. HIF-1α protein levels were normalized to protein levels of β-actin. For quantification of protein expression of mouse proinflammatory cytokines, enzyme-linked immunosorbent assays (ELISAs) were carried out according to manufacturer’s instructions (R&D, Minneapolis, MN, USA). Total soluble protein was measured by Bradford assay according to manufacturer’s instructions (Bio-Rad, Hercules, CA, USA). Protein expression of the inflammatory cytokines was normalized to total soluble protein.

### 2.8. Histological Analysis

For lung histological analyses, formalin-fixed paraffin-embedded lung sections (5 μm) were stained with hematoxylin and eosin (H&E) as previously described [6,29]. The number of tissue-infiltrating cells per pulmonary vessel < 150 µm was counted in H&E-stained lung sections as previously described [4,30].

### 2.9. Statistical Analysis

Pairwise comparisons were assessed with independent student *t*-tests. Multiple group comparisons were assessed using one- or two-way ANOVA with Bonferroni or Sidak’s post-tests, respectively. *p*-values of <0.05 were considered significant.

## 3. Results

### 3.1. Rabeprazole Is an HIF-1α Inducer in Lung Endothelial Cells

**Figure 1:**
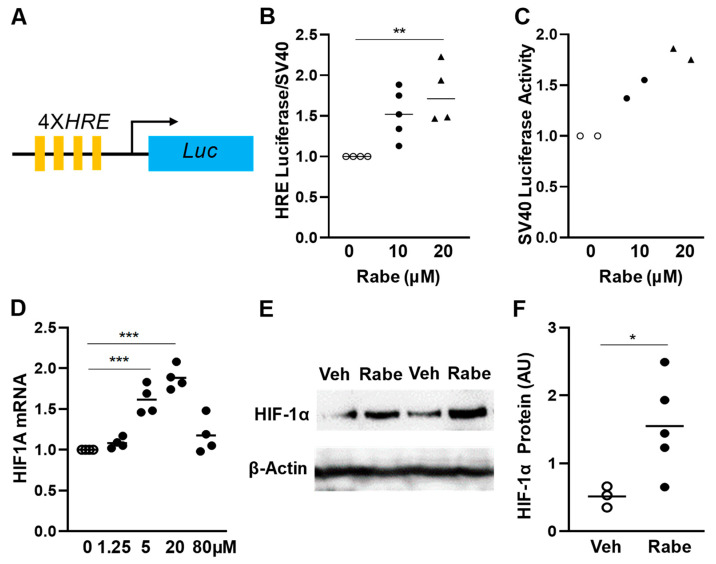
Rabeprazole is a HIF-1α inducer. (**A**) Schematic representation of the plasmid DNA expressing luciferase under the control of 4 hypoxia response elements (4XHRE) in 786-O cells. (**B**,**C**) Validation of Rabeprazole as an HIF-α activator in 786-O cells. Stable 786-O cells with 4XHRE or SV40 promoter luciferase plasmid were seeded in 384-well plates (500 cells/well). Rabeprazole at the indicated concentrations was added to the wells at 24 h after cell seeding. At 48 h later, luminescence signal was measured. Rabeprazole treatment resulted in marked increases in HRE luciferase activity without concomitant decreases in SV40 promoter-controlled luciferase activity, indicating Rabeprazole at the active dose had no toxic effect on the cells. (**D**) Rabeprazole induced HIF1A mRNA in HLMVECs, as shown by RT-qPCR analysis. HLMVECs were seeded into 6-well plates (0.3 × 10^6^ cells/well). At 48 h later, Rabeprazole was added at the indicated concentrations. At 18 h post-treatment, cells were collected for molecular analyses. (**E**,**F**) Rabeprazole treatment increased HIF-1α protein levels in lungs of LPS-challenged WT mice. At 4 and 20 h post-LPS (2.5 mg/kg, i.p.), mice were treated with PBS (Veh) or 20 mg/kg Rabeprazole (Rabe, oral). At 52 h post-LPS, lung tissues were collected for Western blotting (**E**) and quantifications of HIF-1α protein levels (**F**). * *p* < 0.05, ** *p* < 0.01, and *** *p* < 0.001. One-way ANOVA with Bonferroni post-tests (**B**,**D**) or unpaired *t*-test (**F**).

To identify FDA-approved drug(s) that could also activate HIF-α signaling, we carried out high throughput screening of the Prestwick Chemical Library of FDA-approved drugs (1200 compounds) employing the stable HRE/luciferase-expressing human renal cancer cell line, 786-O (Figure 1A). These cells are VHL-deficient, leading to HIF-α stabilization. Rabeprazole (also known as Aciphex) was identified as an HIF-α inducer as evidenced by increases in HRE/luciferase activity (Figure 1B), without obvious toxicity in 786-O cells (Figure 1C). Two other proton pump inhibitors were also screened in our high throughput analysis: Lansoprazole gave rise to a 1.5-fold increase in HRE luciferase activity, while Omeprazole treatment did not alter HRE luciferase activity (data not shown). Given the dose-response activity of Rabeprazole, along with its encouraging toxicity profile, we elected to study the impact of Rabeprazole in our subsequent studies. First, we assessed the impact of Rabeprazole on HIF1A mRNA levels in primary cultures of HLMVECs, showing a dose-response curve with increasing Rabeprazole dose (Figure 1D). Although a higher dose (80 µM) of Rabeprazole failed to induce HIF1A mRNA expression, 20 µM of Rabeprazole induced the highest HIF1A expression. The calculated EC50 of the Rabeprazole induction of HIF1A mRNA expression was 1.7 µM. Next, we assessed the effect of Rabeprazole treatment on HIF-1α protein expression in vivo. In LPS-treated wild type (WT) mice, pulmonary levels of HIF-1α protein were markedly increased by Rabeprazole treatment (Figure 1E,F).

### 3.2. Rabeprazole Treatment Promotes Lung Vascular Repair Following LPS Challenge

**Figure 2:**
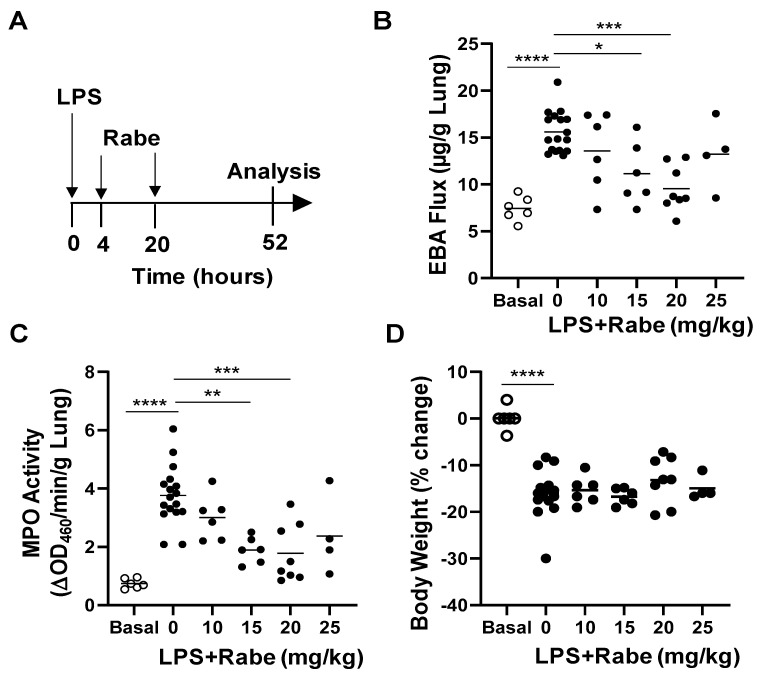
Rabeprazole reduces lung vascular permeability and neutrophil sequestration in a dose-dependent manner at 52 h post-LPS challenge. (**A**) Schematic representation of experimental design. WT mice received PBS vehicle (Basal) or LPS (2.5 mg/kg, i.p.). At 4 and 20 h post-LPS, mice were treated with either PBS vehicle (0 mg/kg) or indicated dose of Rabeprazole (Rabe, oral). Lung tissues were collected for analyses at 52 h post-LPS. (**B**) EBA flux assay showing dose-dependent reductions in lung vascular permeability resulting from oral Rabeprazole treatment. (**C**) Lung neutrophil sequestration as measured by MPO activity assay was reduced in Rabeprazole-treated LPS mice compared to vehicle-treated LPS mice. (**D**) Weight change in LPS-free basal mice and in LPS-challenged mice with or without Rabeprazole treatment. * *p* < 0.05, ** *p* < 0.01, *** *p* < 0.001, and **** *p* < 0.0001. One-way ANOVA with Bonferroni post-tests.

**Figure 3:**
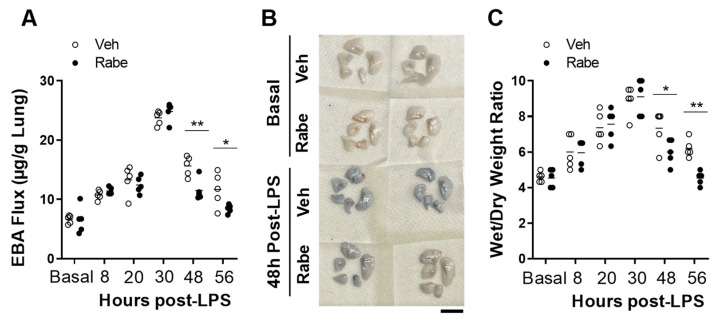
Rabeprazole enhances vascular repair following sepsis-induced inflammatory lung injury. WT mice received PBS vehicle (Basal) or LPS (2.5 mg/kg, i.p.). At 4 and 20 h post-LPS, mice were treated with PBS vehicle or Rabeprazole (18 mg/kg, oral). Lungs were collected at various times post-LPS challenge for EBA flux assay and lung edema assessment. (**A**) EBA flux assay demonstrating that Rabeprazole treatment reduced vascular permeability during the repair phase after LPS challenge but not at baseline or during the injury phase (8–30 h). (**B**) Representative images of lungs from basal mice and LPS-challenged mice at 48 h post-LPS, showing reductions in EBA flux (blue) following treatment with Rabeprazole in LPS-treated mice. Scale bar = 1 cm. (**C**) Lung wet–dry weight ratio demonstrating reductions in lung edema in Rabeprazole- versus vehicle-treated LPS mice during the repair phase but not at baseline or during the injury phase. * *p* < 0.05 and ** *p* < 0.01. Two-way ANOVA with Sidak’s multiple comparisons test.

To determine if Rabeprazole can promote vascular repair following inflammatory lung injury, we carried out dose-response studies in vivo. Endotoxin LPS was administered i.p. to WT mice. At 4 and 20 h later, we administered increasing oral dosages of Rabeprazole (Figure 2A). At 52 h post-LPS, when vascular repair and inflammation resolution programs are underway, we found that both lung vascular permeability (measured by EBA flux assay) and neutrophil sequestration (measured by MPO activity assay) were reduced by Rabeprazole treatment in a dose-dependent manner (Figure 2B,C). None of the doses of Rabeprazole administered to LPS-challenged mice altered body weight loss compared with Rabeprazole-free LPS-challenged mice (Figure 2D). Given that both 15 and 20 mg/kg doses resulted in significant reductions in vascular permeability and MPO activity in the endotoxic lung, we elected to administer a Rabeprazole dose of 18 mg/kg in subsequent studies. In these in vivo studies, WT mice again received LPS challenge followed by oral Rabeprazole at 4 and 20 h after LPS. Rabeprazole treatment did not alter vascular permeability in basal mice (Figure 3A,B). Vascular permeability was unchanged by Rabeprazole treatment during the lung injury phase (i.e., at 8, 20, and 30 h post-LPS) compared to vehicle-treated LPS-challenged mice. However, at the time points of vascular repair and resolution of inflammation (i.e., 48 and 56 h post-LPS), Rabeprazole treatment resulted in marked reductions in vascular permeability (Figure 3A,B). Similarly, Rabeprazole treatment did not alter lung edema (measured by wet–dry lung weight ratio) in basal mice (Figure 3C). Lung edema was also unchanged by Rabeprazole treatment during the lung injury phase (i.e., at 8, 20, and 30 h post-LPS). However, at 48 and 56 h post-LPS, Rabeprazole treatment resulted in reductions in lung edema (Figure 3C). Together, these data demonstrate that Rabeprazole has no effects on lung vascular integrity at basal or during the injury phase following LPS challenge, whereas it promotes vascular repair during the reparative phase.

### 3.3. Rabeprazole Treatment Augments Resolution of Lung Inflammation after LPS Challenge

**Figure 4:**
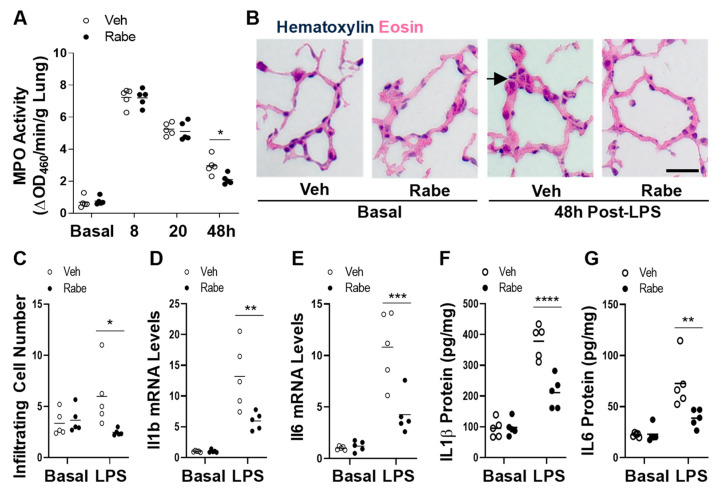
Rabeprazole promotes resolution of inflammation following sepsis challenge. (**A**) MPO activity assay showing Rabeprazole treatment resulted in reduced neutrophil sequestration during the repair phase (e.g., 48 h) after LPS challenge but not at baseline or during the injury phase (8 and 24 h). WT mice received PBS vehicle (Basal) or LPS (2.5 mg/kg, i.p.). At 4 and 20 h post-LPS, mice were treated with PBS vehicle or Rabeprazole (18 mg/kg, oral). (**B**) Representative images of infiltrating peri-vascular cells in H&E-stained lungs from basal and LPS-challenged mice at 48 h post-LPS. Arrow indicates infiltrated peri-vascular cells. (**C**) Quantifications of infiltrated proinflammatory cells per vessel < 150 µm. (**D**,**E**) Quantitative RT-PCR and (**F**,**G**) ELISA analyses demonstrating Rabeprazole treatment inhibited expression of proinflammatory cytokines at 48 h post-LPS but not at baseline. Scale bar = 50 μm. * *p* < 0.05, ** *p* < 0.01, *** *p* < 0.001, and **** *p* < 0.0001. Two-way ANOVA with Sidak’s post-tests.

We next determined whether Rabeprazole treatment accelerated the resolution of lung inflammation. As shown in Figure 4A, Rabeprazole treatment did not alter neutrophil sequestration in the lung (measured by MPO activity assay) at baseline or during the injury phase following LPS challenge. At 48 h post-LPS, however, Rabeprazole treatment resulted in reduced MPO activity compared with the vehicle group. Accordingly, histological examination also revealed less proinflammatory cell sequestration in Rabeprazole-treated LPS mice compared to vehicle-treated LPS mice (Figure 4B,C). We also determined the expression of proinflammatory genes, interleukin (IL) 1β and IL6. Quantitative RT-PCR analysis demonstrated marked reductions in *Il1b* and *Il6* in the lungs of Rabeprazole-treated mice at 48 h post-LPS compared to vehicle-treated LPS mice (Figure 4D,E). However, there was no difference between Rabeprazole- and vehicle-treated LPS mice at baseline. ELISAs showed that the protein levels of these two inflammatory cytokines (IL1β and IL6) followed similar expression patterns as the mRNA expression levels (Figure 4F,G) Together, these data showed that Rabeprazole treatment accelerates the resolution of sepsis-induced lung inflammation.

### 3.4. Rabeprazole Increases HIF-1α/FoxM1 Signaling after LPS Challenge

**Figure 5:**
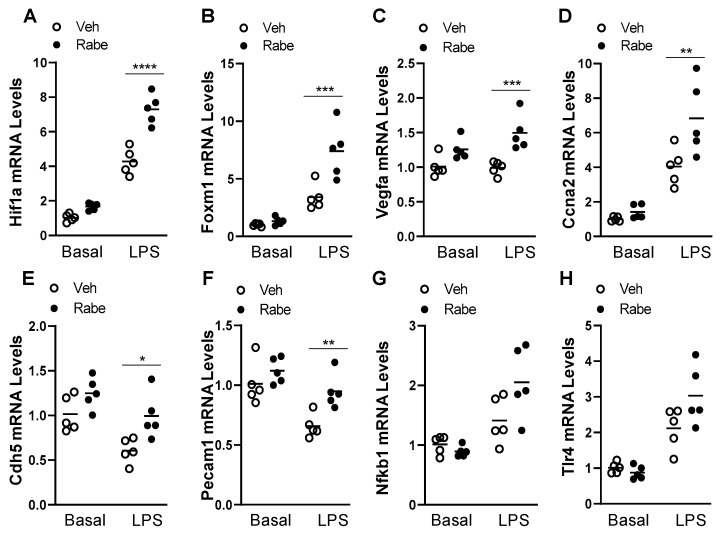
Rabeprazole promotes HIF1α/FoxM1 signaling after LPS challenge. (**A**–**H**) WT mice received PBS vehicle (Basal) or LPS (2.5 mg/kg, i.p.). At 4 and 20 h post-LPS, mice were treated with PBS vehicle (Veh) or Rabeprazole (Rabe, 18 mg/kg, oral). At 48 h post-LPS, lung tissues were collected for RNA isolation and quantitative RT-PCR analyses of various genes. * *p* < 0.05, ** *p* < 0.01, *** *p* < 0.001, and **** *p* < 0.0001. Two-way ANOVA with Sidak’s post-tests.

To explore the possible mechanisms through which Rabeprazole acts in vivo, we next assessed the levels of factors that are involved in the reparative HIF-1α/FoxM1 signaling pathway, which we previously found to be responsible for vascular repair and the resolution of inflammation in mouse models of endotoxemic and polymicrobial sepsis-induced lung injury [6]. We performed quantitative RT-PCR analysis of whole lung samples from LPS-free basal mice and at 48 h after LPS. In LPS-challenged mice, Rabeprazole treatment expectedly increased the expression of Hif1a, as well as its proliferative downstream targets, Foxm1, and vascular endothelial growth factor (Vegfa), without altering levels in basal mice (Figure 5A–C). In the lungs of LPS-treated but not LPS-free mice, there were also Rabeprazole-induced increases in cell cycle regulator cyclin A2 (Ccna2), a transcriptional target of FoxM1 (4), and the EC junction molecules, platelet and endothelial cell adhesion molecule 1 (Pecam1, a.k.a. CD31) and cadherin 5 (Cdh5, a.k.a. VE-Cadherin) (Figure 5D–F). Previous studies have assessed the impact of proton pump inhibitors on the nuclear factor kappa B (NFkB) and toll-like receptor 4 (TLR4) signaling pathway in gastric epithelial and cancer cells, kidney epithelial and tubular cells, monocytes, umbilical vein ECs, and glioma cells [31,32,33,34,35]. In our study, whole lung levels of nuclear factor kappa B (NFkb1) and toll-like receptor 4 (Tlr4) were not significantly altered by Rabeprazole treatment in basal or sepsis-challenged mice (Figure 5G,H). These data together support the possibility that Rabeprazole treatment increases vascular repair and the resolution of inflammation through EC-proliferative signaling and endothelial junction reannealing that is driven by HIF-1α/FoxM1.

### 3.5. Rabeprazole Enhances Vascular Repair and Resolution of Sepsis-Induced Lung Inflammation through Endothelial HIF-1α

**Figure 6:**
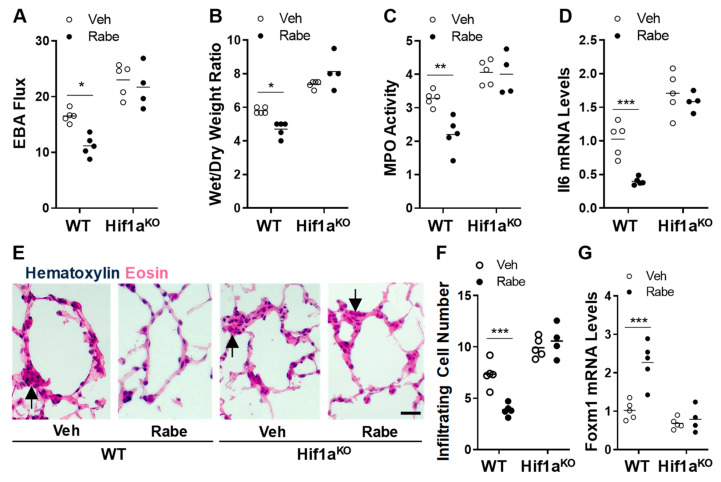
Rabeprazole enhances vascular repair and resolution of sepsis-induced inflammatory lung injury through HIF-1α. WT or *Hif1a/Tie2*Cre knockout (Hif1a^KO^) mice received LPS (2.5 mg/kg, i.p.). At 4 and 20 h post-LPS, mice were treated with PBS vehicle or Rabeprazole (18 mg/kg, oral). Lungs were analyzed at 52 h post-LPS. (**A**) EBA flux assay demonstrating that Rabeprazole treatment-induced decreases in vascular permeability seen in WT mice were absent in conditional Hif1a/Tie2Cre KO mice. (**B**) Rabeprazole-induced reduction in lung edema measured by lung wet/dry weight ratio was dependent upon HIF-1α. (**C**) MPO activity assay showing that Rabeprazole reduces neutrophil sequestration in lungs of WT but not Hif1a/Tie2Cre KO mice. (**D**) Pulmonary expression of proinflammatory cytokine Il6 was reduced by Rabeprazole treatment in WT but not Hif1a/Tie2Cre mice. (**E**) Representative images of infiltrating peri-vascular cells in H&E-stained lungs from Rabeprazole-treated and Rabeprazole-free WT and Hif1a/Tie2Cre mice at 52 h post-LPS. Arrows indicate infiltrated peri-vascular cells. Scale bar = 50 μm. (**F**) Quantifications of infiltrated proinflammatory cells per vessel < 150 µm. (**G**) Pulmonary FoxM1 expression was increased by Rabeprazole treatment in an HIF-1α-dependent manner. * *p* < 0.05, ** *p* < 0.01, and *** *p* < 0.001. Two-way ANOVA with Sidak’s post-tests.

To assess whether Rabeprazole improved vascular repair and the resolution of inflammation through HIF-1α, we assessed the vascular repair and inflammation resolution in mice with or without conditional deletion of *Hif1a* driven by *Tie2*Cre [6]. We found that the Rabeprazole-dependent reductions in vascular permeability, edema, MPO activity, *Il6* expression, and peri-vascular cell infiltration during the repair phase seen in LPS-challenged WT mice were totally absent in LPS-challenged *Hif1a/Tie2*Cre knockout mice (Figure 5A–F). Consistent with our previous study in *Hif1a/Tie2*Cre knockout mice [6], we also observed impaired vascular repair and resolution of inflammation in vehicle-treated *Hif1a/Tie2*Cre knockout mice compared to vehicle-treated WT mice at 52 h post-LPS (Figure 6A–F). Together, these data demonstrate that Rabeprazole is an HIF-1α inducer in vivo, which promotes vascular repair and the resolution of inflammation in an HIF-1α-dependent manner.

### 3.6. FoxM1 Is the Downstream Transcriptional Factor of HIF-1α Mediating Rabeprazole-Induced Reparative Effects

Our previously published study shows that FoxM1 is the downstream target of HIF-1α that is responsible for vascular repair following sepsis challenge [6]. We next determined if Rabeprazole can activate FoxM1 expression in an HIF-1α-dependent manner. Quantitative RT-PCR analysis showed a marked induction of *Foxm1* expression in WT mice at 52 h post-LPS challenge compared to vehicle-treated WT mice (Figure 6G). However, *Foxm1* expression was not induced in the lungs of Rabeprazole-treated *Hif1a/Tie2*Cre KO mice, demonstrating that Rabeprazole-induced FoxM1 expression is dependent upon HIF-1α.

**Figure 7:**
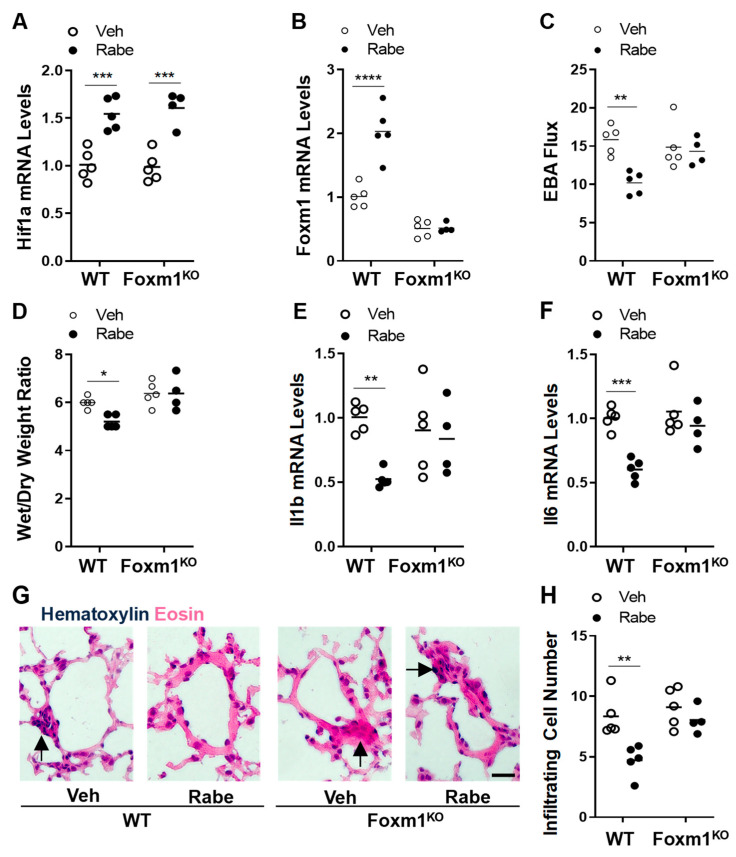
Genetic disruption of FoxM1 abrogates the reparative effects of Rabeprazole following sepsis-induced inflammatory lung injury. (**A**) Quantitative RT-PCR analysis of HIF-1α and (**B**) FoxM1 expression in mouse lungs. WT or conditional *Foxm1/Tie2*Cre (Foxm1^KO^) mice received LPS (2.5 mg/kg, i.p.). At 4 and 20 h post-LPS, mice were treated with PBS vehicle or Rabeprazole (18 mg/kg, oral). Lungs were analyzed at 52 h post-LPS. (**C**) EBA flux assay demonstrating that Rabeprazole-dependent decreases in lung vascular permeability seen in WT mice were absent in *Foxm1/Tie2*Cre mice. (**D**) Lung edema measured by lung wet–dry weight ratio was not inhibited in Rabeprazole-treated *Foxm1/Tie2*Cre mice in contrast to Rabeprazole-treated WT mice. (**E**) Quantitative RT-PCR analysis of Il1b and (**F**) Il6 demonstrating that Rabeprazole-dependent decreases in lung inflammation seen in WT mice were absent in Foxm1/Tie2Cre mice. (**G**) Representative images of infiltrating peri-vascular cells in H&E-stained lungs from Rabeprazole-treated and Rabeprazole-free WT and Foxm1/Tie2Cre mice at 52 h post-LPS. Arrows indicate infiltrated peri-vascular cells. Scale bar = 50 µm. (**H**) Quantifications of infiltrated proinflammatory cells per vessel < 150 µm. * *p* < 0.05, ** *p* < 0.01, *** *p* < 0.001, and **** *p* < 0.0001. Two-way ANOVA with Sidak’s post-tests.

Given that endothelial FoxM1 is a critical reparative transcriptional factor mediating vascular repair following sepsis challenge [4,5,28,36], we next determined the effects of Rabeprazole treatment in *Foxm1/Tie2*Cre mice. Quantitative RT-PCR analysis showed that whole lung *Hif1a* mRNA expression was increased by Rabeprazole treatment in WT and *Foxm1/Tie2*Cre mice (Figure 7A). Quantitative RT-PCR analysis also demonstrated a marked decrease in *Foxm1* expression in the lungs of *Foxm1/Tie2*Cre mice compared to WT mice, confirming *Foxm1* deletion (Figure 7B). Rabeprazole-induced *Foxm1* expression in WT mice was also abrogated in *Foxm1/Tie2*Cre mice (Figure 7B). These data also indicate Rabeprazole-induced FoxM1 expression is predominantly in pulmonary vascular ECs. An EBA flux assay showed that Rabeprazole-induced decreases in vascular permeability in WT mice were largely diminished in *Foxm1/Tie2*Cre mice at 52 h post-LPS challenge (Figure 7C). Accordingly, Rabeprazole-treated *Foxm1/Tie2*Cre mice exhibited lung edema similar to vehicle-treated WT mice, which was in contrast to Rabeprazole-treated WT mice (Figure 7D). Rabeprazole-dependent decreases in proinflammatory cytokine expression and peri-vascular cell infiltration that were present in WT mice were also absent in *Foxm1/Tie2*Cre mice (Figure 7E–H). These data together show that Rabeprazole enhances vascular repair and the resolution of inflammatory lung injury through FoxM1.

## 4. Discussion

In this study, we show that Rabeprazole is a HIF-1α inducer that accelerates pulmonary vascular repair and the resolution of lung inflammation in a mouse model of endotoxic sepsis. Rabeprazole is an oral proton pump inhibitor that is used in the clinic to treat stomach and esophagus problems such as acid reflux and ulcers [37,38,39]. We administered oral Rabeprazole to mice with sepsis-induced inflammatory lung injury and at doses that are equivalent to those well tolerated in humans. Rabeprazole was administered to mice that had already received an endotoxic sepsis challenge; this reparative treatment accelerated vascular repair and the resolution of inflammation. In *Hif1a/Tie2*Cre knockout mice, Rabeprazole failed to induce FoxM1 expression and promote vascular repair and the resolution of inflammation. Similarly, Rabeprazole was not effective in activating vascular repair in *Foxm1/Tie2*Cre knockout mice, further demonstrating that the mechanism of action of Rabeprazole is through HIF-1α/FoxM1 signaling.

HIF-1α is rapidly induced and stabilized in mouse lung vascular ECs after sepsis challenge [6]. Mice with EC-specific KO of *Hif1a* (*Hif1a/Tie2*Cre) exhibit impaired lung endothelial regeneration and vascular repair in contrast to wild type mice, despite similar levels of peak injury following sepsis challenge [6]. Restoration of FoxM1 expression in ECs of these EC-specific *Hif1a* KO mice normalized endothelial proliferation and vascular repair, demonstrating that pulmonary vascular endothelial HIF-1α is required for lung endothelial regeneration and vascular repair via FoxM1 after sepsis-induced lung injury [6]. In another study of HIF-1α signaling during post-sepsis vascular repair in mouse lungs, promoter analysis identified Sox17 as a reparative transcriptional target of HIF-1α [40]. These studies demonstrate the critical role of HIF-1α in mediating lung vascular repair and the resolution of inflammation following sepsis challenge. Thus, the identification of an existing HIF-1α inducer/activator has great clinical potential for the treatment of severe sepsis and ARDS. Other studies also demonstrate an important role of HIF-1α in vascular repair in animal models other than sepsis. In a mouse orthotopic tracheal transplant model, topical application of the HIF-1α inducer, deferoxamine, to the transplants results in increased lung EC proliferation and decreased apoptosis [41]. In another study of airway microvascular regeneration, VHL-haplodeficient (leading to stabilization of HIF-α) airway ECs exhibit enhanced microvascular repair as shown by increased EC survival and migration [42]. Conversely, in transplant recipient mice with *Hif1a* deletion in *Tie2* positive cells, microvascular repair is impaired [42]. As well as EC-specific HIF-1α, epithelial HIF-1α has also been shown to promote alveolar proliferation and repair through the HIF-1 target, stromal cell-derived factor (SDF) 1 and its receptor, chemokine-X-chemokine receptor (CXCR) 4 [43]. Furthermore, PHD1- and PHD3-deficient mice show increased HIF-1α expression in pulmonary neuroepithelial bodies, along with increased expression of the proliferation marker, Ki67 [44].

Our study here provides unequivocal evidence that Rabeprazole is a HIF-1α inducer, which promotes vascular repair and the resolution of inflammation via endothelial HIF-1α following sepsis challenge. Furthermore, we also show that Rabeprazole treatment resulted in a marked increase in FoxM1 expression in LPS-challenged WT mice, which was, however, blocked in *Hif1a/Tie2*Cre mice. Employing *Foxm1/Tie2*Cre mice, we demonstrated that Rabeprazole-induced FoxM1 expression was predominantly in lung ECs following sepsis challenge and the induced FoxM1 expression was required for Rabeprazole-induced vascular repair. Taken together, these data demonstrate a novel role of Rabeprazole in promoting vascular repair following inflammatory lung injury through the activation of HIF-1α/FoxM1 signaling in ECs. Thus, Rabeprazole may be repurposed for the treatment of severe sepsis and ARDS. It may also be worthwhile to test its therapeutic potential in patients with severe COVID-19, as endothelial injury is a characteristic feature of COVID-19 lung injury [45,46,47,48], and COVID-19 ARDS is considered as a vascular endotype of ARDS [49,50,51]. Vadadustat, an HIF prolyl hydroxylase inhibitor, which stabilizes HIF-α [52], is currently under phase II clinical trial for the prevention and treatment of ARDS in hospitalized COVID-19 patients (NCT04478071). It will be interesting to see whether Vadadustat is effective in treating severe COVID-19 patients.

Our finding that Rabeprazole induces HIF-1α expression is consistent with a large database of gene expression data from multiple cell culture studies, showing that Rabeprazole treatment leads to an 8.5-fold increase in HIF-1α expression in human liver hepatocytes (ToxDB Database) [53]. When we assessed our high throughput screening data of 786-O cells, we found that while one other proton pump inhibitor (i.e., Lansoprazole) also increased HRE luciferase activity, another proton pump inhibitor (i.e., Omeprazole) did not alter HRE luciferase activity. A previous study in gastric cancer cells showed that Omeprazole treatment reduces HIF-1α expression [54]. Another proton pump inhibitor, Pantoprazole, was also shown to downregulate the HIF-1α signaling pathway in human gastric adenocarcinoma cells [55]. Given the divergent effects of different proton pump inhibitors on HIF-1 signaling, it can be proposed that the impact of Rabeprazole on HIF-1α signaling is an individual drug action. Our study does not, however, fully elucidate the mechanism by which Rabeprazole acts as an inducer of HIF-1α in ECs. The mechanism of action of Rabeprazole on HIF-1α expression and/or HIF-1 activation should be investigated in future studies.

Rabeprazole treatment improved vascular repair and the resolution of inflammation but did not alter peak vascular injury or inflammation. It is therefore highly likely that Rabeprazole improves components of vascular repair and the resolution of inflammation such as EC proliferation and EC–EC junction reannealing, without altering components of lung injury such as EC death and EC–EC junction weakening. Given that HIF-1 targets include multiple reparative and proliferative factors [20,56,57,58,59], and that Rabeprazole treatment increased the expression of proliferative HIF-1 targets such as FoxM1 and VEGF in whole lung samples after LPS challenge, we propose that Rabeprazole- and HIF-1α-dependent increases in vascular repair are due to increases in the expression of reparative HIF-1α targets such as FoxM1 [6] and VEGF [60], which lead to downstream increases in vascular repair. In support of this possibility, we have previously shown that the HIF-1α/FoxM1 signaling axis increases vascular repair and the resolution of inflammation through increases in EC proliferation and regeneration and EC–EC reannealing [4,6,28].

Our study has shown that Rabeprazole treatment has no effect on lung vascular permeability and neutrophil sequestration at base line and also during the injury phase following LPS challenge, demonstrating the unique role of Rabeprazole in activating vascular repair and the resolution of inflammation. Gong et al. showed that HIF-2α plays an important role in inhibiting adherens junctional disruption in acute lung injury by inducing VE-PTP expression, which reduces VE-Cadherin endocytosis by enhancing VE-cadherin dephosphorylation, and thus augments endothelial adherens junction integrity and endothelial barrier function [61]. Mice with endothelial disruption of *Hif2a* exhibited increased lung vascular permeability at base line and during the injury phase (e.g., at 12 h) following LPS challenge [61]. The lack of effects of Rabeprazole on lung vascular permeability at base line and during the injury phase following LPS challenge further support the concept that Rabeprazole functions through HIF-1α not HIF-2α. Recent studies have shown the detrimental effects of HIF-2α activation in inducing renal cancer and pulmonary arterial hypertension. Both Vadadustat and Roxadustat [62] are two clinically used pan-HIF-α agonists that act through inhibition of HIF prolyl hydroxylase-mediated degradation; their potential off-target effects and side effects may hamper their use for the treatment of severe sepsis and ARDS. Selective activation of HIF-1α may be an alternative effective and relatively safe approach for treatment of severe sepsis and ARDS.

## 5. Conclusions

In summary, our study has for the first time identified Rabeprazole as a HIF-1α inducer. Rabeprazole improves vascular repair and the resolution of sepsis-induced inflammatory lung injury through the endothelial HIF-1α/FoxM1 signaling axis. FDA-approved drugs that stimulate HIF-1α/FoxM1 signaling, such as Rabeprazole, represent promising candidate therapies for patients with severe sepsis and ARDS. Our study provides further evidence that vascular repair and the resolution of inflammatory lung injury is driven by HIF-1α and FoxM1 in ECs and that pharmacological activation of the HIF-1α/FoxM1 signaling axis is a putative therapeutic strategy for severe sepsis and ARDS.

## Data Availability

The data presented in this study are available on request from the corresponding author(s).
